# In vitro antitumor, pro-inflammatory, and pro-coagulant activities of *Megalopyge opercularis* J.E. Smith hemolymph and spine venom

**DOI:** 10.1038/s41598-020-75231-1

**Published:** 2020-10-27

**Authors:** Alonso A. Orozco-Flores, José A. Valadez-Lira, Karina E. Covarrubias-Cárdenas, José J. Pérez-Trujillo, Ricardo Gomez-Flores, Diana Caballero-Hernández, Reyes Tamez-Guerra, Cristina Rodríguez-Padilla, Patricia Tamez-Guerra

**Affiliations:** 1 Departamento de Microbiología E Inmunología, Facultad de Ciencias Biológicas (FCB), Universidad Autónoma de Nuevo León (UANL), Cd. Universitaria, AP. 46-F., 66455 San Nicolás de los Garza, NL Mexico; 2 Departamento de Histología, Facultad de Medicina, UANL, Monterrey, NL Mexico

**Keywords:** Chemokines, Coagulation system, Cytokines, Haematopoiesis, Immunotherapy, Tumour immunology, Ecology, Immunology

## Abstract

Contact with stinging spines venom from several Lepidoptera larvae may result in skin lesions. In Mexico, envenomation outbreaks caused by *Megalopyge opercularis* were reported between 2015 and 2016. The aim of this study was to identify the venomous caterpillars in Nuevo Leon, Mexico and evaluate several biological activities of their hemolymph (HEV) and spine setae (SSV) venoms. *M. opercularis* was identified by cytochrome oxidase subunit (COI) designed primers. HEV and SSV extracts cytotoxic activity was assessed on the L5178Y-R lymphoma cell line. For apoptotic cells number and apoptosis, cells were stained with acridine orange/ethidium bromide and validated by DNA fragmentation. Human peripheral blood mononuclear cells (hPBMC) cytokine response to the extracts was measured by the cytometric bead array assay. Extracts effect on pro-coagulation activity on human plasma was also evaluated. HEV and SSV extracts significantly inhibited (*p* < 0.01) up to 63% L5178Y-R tumor cell growth at 125–500 µg/mL, as compared with 43% of Vincristine. About 79% extracts-treated tumor cells death was caused by apoptosis. Extracts stimulated (*p* < 0.01) up to 60% proliferation of resident murine lymphocytes, upregulated IL-1β, IL-6, IL-8, and TNF-α production by hPBMC, and showed potent pro-coagulant effects. The pharmacological relevance of these venoms is discussed.

## Introduction

Lepidoptera order consists of moths and butterflies distributed throughout almost the entire planet. About 12 families have poisonous species associated with human skin inflammatory responses^1^. Most of them belong to the Saturniidae, Limacodidae, Megalopygidae, Arctiidae, Lasiocampidae, Notodentidae, and Lymantridae families^2–4^. One of the most important Megalopygidae species is represented by the *Megalopyge* genera^5^. Larvae of these genera have spines, which contain a high number of specialized cells with a sensory function and potential to respond to mechanical stimulation by secreting and injecting toxins into the skin through fur-like spines^6^. Envenomation caused by contact with *Megalopyge opercularis* spines causes severe pain, burning, and swelling, in addition to nausea and headache symptoms.

In mid-November of 2015 and October of 2016, increased skin injury alerts after venomous puss caterpillar (“oruga peluche”) stings were reported, mainly among infants, in the Mexican states of Jalisco, Nuevo León, and Tamaulipas (https://www.milenio.com/region/Reportan_presencia_oruga_peluchecasos_oruga_peluche_NL-alertan_oruga_peluche_0_630537166.html). In the USA, *M. opercularis* envenomation period ranged from July to November, but higher case numbers were reported from September to November^7^. Among others, the puss caterpillar, with a characteristic “hairy fur-like” appearance resembling a Persian cat, represents the insect’s larval stage, whereas *M. opercularis* adults are known as southern flannel moths^7^. This species is the most widely distributed of the genus, being reported from the southern states of the USA to South America^8^. Other *Megalopyge* species, including *M. urens, M. lanata,* and *M. krugi* from Central and South America, have the potential to cause severe stings^9,10^. Therefore, taxonomic identification of venomous caterpillar species at the outbreak location is ecologically and epidemiologically relevant^11^.

Because of its appearance, the puss caterpillar is very alluring to touch, particularly by children. Unfortunately, hidden in its furry hair, the larva has hollow spines ended in setae. After contact, a sensory and mechanical response activates spines insertion and toxin secretion into the skin^12^. Affected individuals acquire the venom from each broken setae, thus increasing the injury in the skin and surrounded area^3,8^.

The insect’s advantage of producing such poisoning compounds, in addition to avoid predation by other animals, may be related to improve its cellular longevity by inhibiting apoptosis^11,13–16^, thus showing application in biotechnology, mainly in the clinical area.

Evaluating *M. opercularis* venom symptoms in the Central Texas Poison Center during 1996, almost 99% of patients experienced local pain, 27% referred intense radiating pain and presented edema, 42% developed erythema, and 9% had welts, among other symptoms^17^. These varying symptoms among humans exposed to *M. opercularis* venom may be an indication of differences in their immune responses.

Compounds with venom-like properties have been isolated from the spine setae and hemolymph extracts of stinging caterpillars^11^. Such agents may consist of peptidic and non-peptidic components, however, their full nature and activities have not been fully investigated^11^. Recently, Sánchez et al. reported that *M. lanata* peptidic venom induced mild skin inflammatory lesions in mice and elicited a limited shortening clotting time triggered by calcium^17^.

In addition, spine structures produce and inoculate chemical urticating compounds. They are filled with a fluid that is secreted by cells located at the base of the tegument^3^. It has been suggested that spine cells are responsible for the venom secretion by the South America specie *Lonomia obliqua* Walker (Lepidoptera: Saturnidae), which has structures involved in venom production located in the caterpillar body’s proximity and in the hemolymph^8^.

In regard to megalopygids toxicity, Sánchez et al.^18^ showed that proteins found in the species *Megalopyge lanata* (Stoll) and *Podalia orsilochus* (Cramer) have serine peptides similar to those reported by the urticating setae of the neotropical moth *Hylesia metabus* (Cramer), as well as other enzymes reported by *Lenomia obliqua*. In fact, they discussed that such proteins have very low activity towards hyaluronic acid, which is related to venoms, since > 45 kDa hyaluronidases are associated with extracellular matrix components degradation. Although they did not identify the predominant infiltrating mononuclear cell type in skin lesions upon *M. lanata* venom exposure, after testing the spine setae extracts in vivo using a murine model, such injuries were similar to those reported by *L. obliqua* venom, since in vivo cell reaction against *M. opercularis* venom was mostly lymphocytic.

In regard to antitumor properties of insect toxins and proteins, *Apis mellifera* L. (Apidae: Hymenoptera) bee venom (also known as api-toxin) contains phospholipases (also found in *L. obliqua* venom) and melittin peptide, which demonstrated cytotoxic activity against several tumor cell lines, where the melittin-induced anticancer effect was mediated by apoptosis^19^. Furthermore, Suttmann et al. reported that cecropins A and B inhibited bladder cancer cells growth, but were non-toxic against fibroblasts^20^. In contrast, Mazzoni et al. reported an anti-apoptotic effect in VERO and Sf-9 cells of a protein isolated from the close relative *Megalopyge albicollis* Walker hemolymph^21^.

The aim of the present study was to investigate in vitro antitumor, pro-inflammatory, and pro-coagulant activities of *M. opercularis* hemolymph (HEV) and spine setae (SSV) extracts, which may prompt pre-clinical studies for application in biomedicine.

## Results

### Venomous caterpillar identification

The analysis of the COI gene sequence (https://boldsystems.org/index.php/Taxbrowser_Taxonpage?taxon=+Megalopyge&searchMenu=taxonomy&query=+Megalopyge) confirmed that collected larvae belonged to *M. opercularis* species (GenBank access No. MF621253). This is the first molecular identification description of this species in Mexico, according to the Barcode of Life Data Systems (BOLDSYSTEMS v3 database, https://v3.boldsystems.org/index.php/Public_SearchTerms).

In the present study, fresh and washed oak leaves (*Q. virginiana fusiformis*) were used to feed collected larvae and maintain their natural wild type diet. Although all rearing larvae were eating, only six reached the pupal stage and from these, only 2 adults emerged, which may be related to sub-optimal environmental conditions present in the insect colony rearing room; in nature, they seek for colder, tree-shaded outdoor recreation areas during warmer fall and spring months, which synchronizes with peak larval instar seasons^7^.

### Antitumor activity of *M. opercularis* extracts

**Figure 1:**
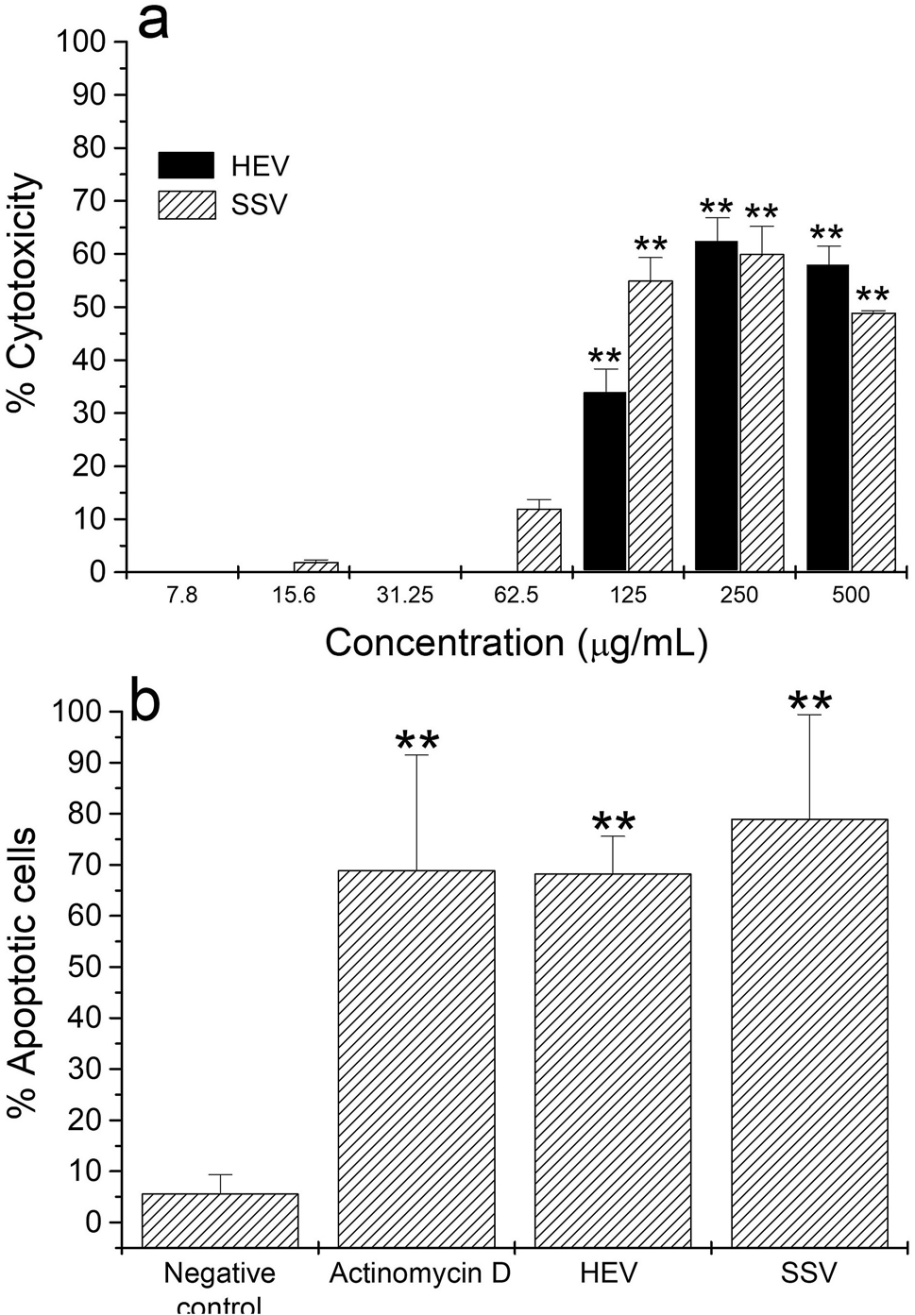
Tumor cell cytotoxicity and apoptosis induced by HEV and SSV extracts. Exponentially growing L5178Y-R cells (5 × 10^4^ cells/mL) were incubated for 48 h in the presence or absence of 7.8–500 µg/mL of HEV and SSV extracts, as described in the text. After incubation for 44 h, MTT was added to all wells, and cultures were incubated for additional 4 h. (**a**) Optical densities at 540 nm and percent cytotoxicity were then determined, as explained in the text. Data represent means ± SD of triplicate determinations from three independent experiments. Optical density value for untreated control was 0.269 ± 0.004. **p < 0.01, compared with untreated control. (**b**) L5178Y-R culture of 3 × 10^6^ cells were exposed to HEV, SSV, or Actinomycin D (positive control), and number of apoptotic cells, were evaluated, as described in the text.

*M. opercularis* HEV and SSV extracts significantly (*p* < 0.01) inhibited 35%, 63%, and 60%, and 56%, 61%, and 51% L5178Y-R growth at 125, 250, and 500 µg/mL, respectively (Fig. 1a), whereas Vincristine (positive control) caused from 32 to 43% tumor growth inhibition at concentrations ranging from of 3.1 to 125 µg/mL^15,37^. For HEV, the IC_95_ was 210.15 µg/mL (± 97.76 to 377.47 µg/mL), whereas for SSV, the IC_95_ was 308.65 µg/mL (± 188.70 to 539.40 µg/mL). The lymphoma cytotoxicity peak was observed at 250 μg/mL (Fig. 1a).

Apoptosis assay was examined after 24 h to achieve an intermediate point of cells toxicity, which was observed after 48 h. Figure 1b shows that 68% and 79% HEV- and SSV-treated tumor cells, respectively, resulted in death by apoptosis (DNA ladder is shown in Supplementary Fig. 1), as compared with 69% of Actinomycin D control-treated cells.

### Effect of HEV and SSV on T cell proliferation

**Figure 2:**
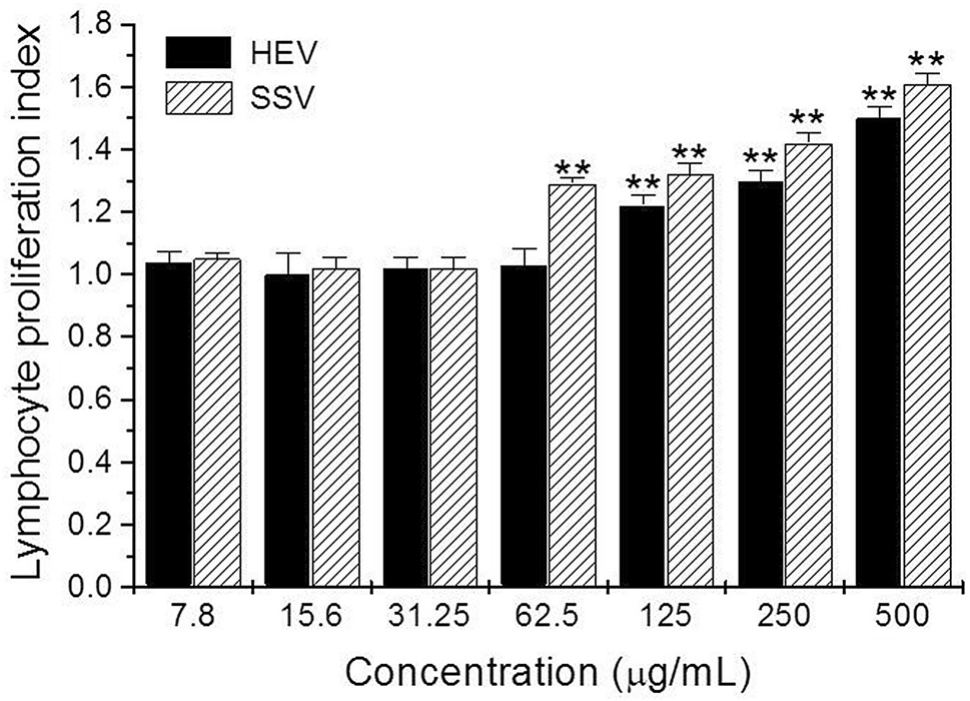
Lymphoproliferation induced by HEV and SSV extracts. Thymus cell suspensions (100 µL of 1 × 10^7^ cells/mL) were incubated in the presence or absence of HEV and SSV at various concentrations, and proliferation assessed as described in the text. Data represent means ± SD of triplicate determinations from three independent experiments. **p < 0.01, compared with untreated control. Optical density at 540 nm for untreated cells was 0.95 ± 0.01. Lymphocyte proliferation index of 1 indicates no alteration on proliferation.

HEV and SSV extracts significantly (*p* < 0.01) stimulated 22–50% and 32–60% resident lymphocytes proliferation from 62.5 µg/mL respectively, as compared with positive control Concanavalin A (6.25 μg/mL) (Fig. 2).

### hPBMC cytokine response to *M. opercularis* extracts

**Figure 3:**
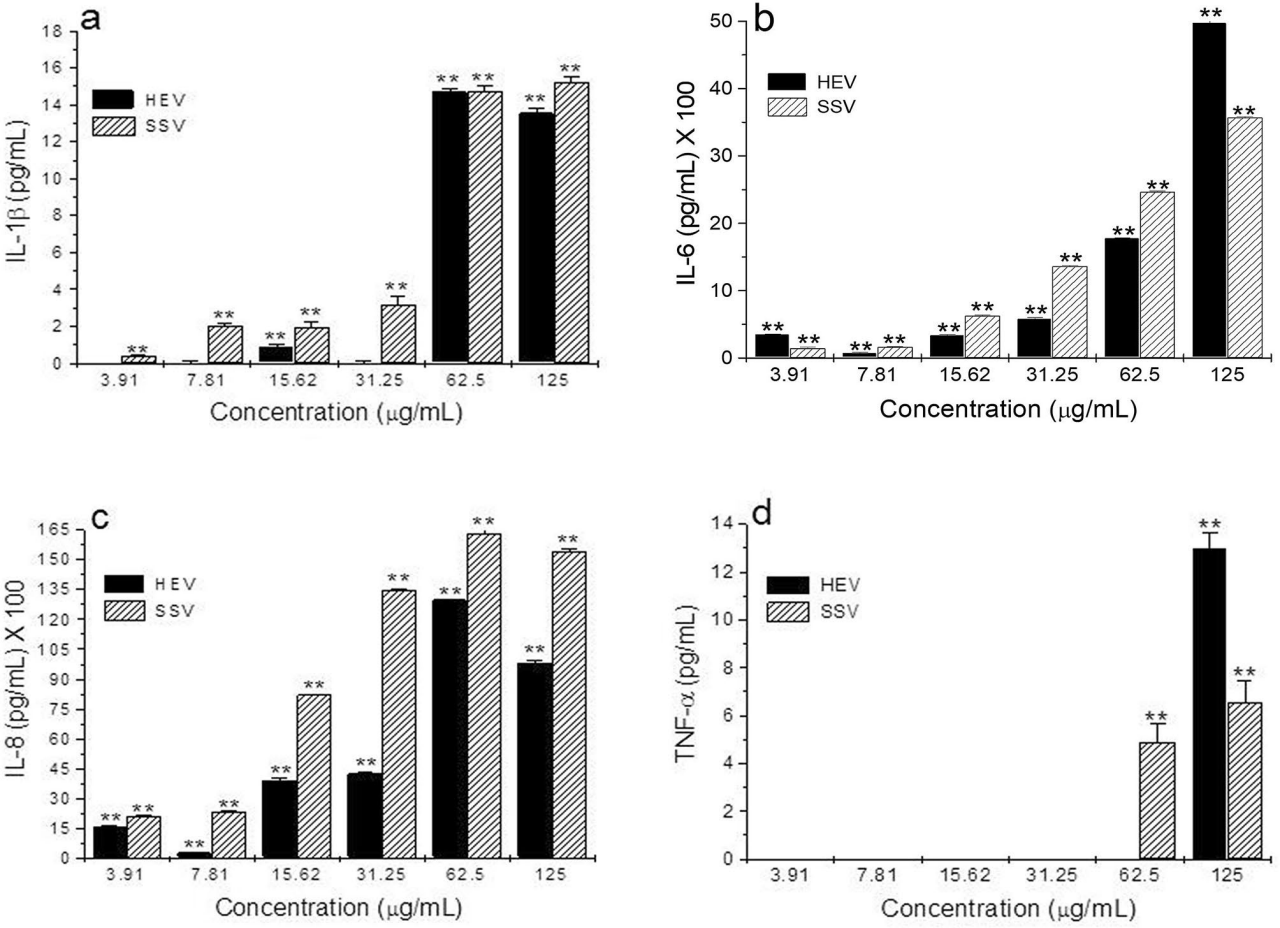
Effect of HEV and SSV on pro-inflammatory cytokine production. (**a**) IL-1β, (**b**) IL-6, (**c**) IL-8, and (**d**) TNF-α production by human PBMC exposed to HEV and SSV extracts at concentrations ranging from 3.91 to 125 µg/mL, was evaluated, as described in the text. Data represent means ± SD of triplicate determinations from three independent experiments. **p < 0.01, compared with untreated control.

As shown in Fig. 3, HEV and SSV extracts increased IL-1β, IL-6, IL-8, and TNF-α cytokines production by hPBMC in a concentration-dependent fashion, as compared with untreated control (undetectable cytokine levels).

### Pro-coagulation effect of HEV and SSV on human plasma

**Figure 4:**
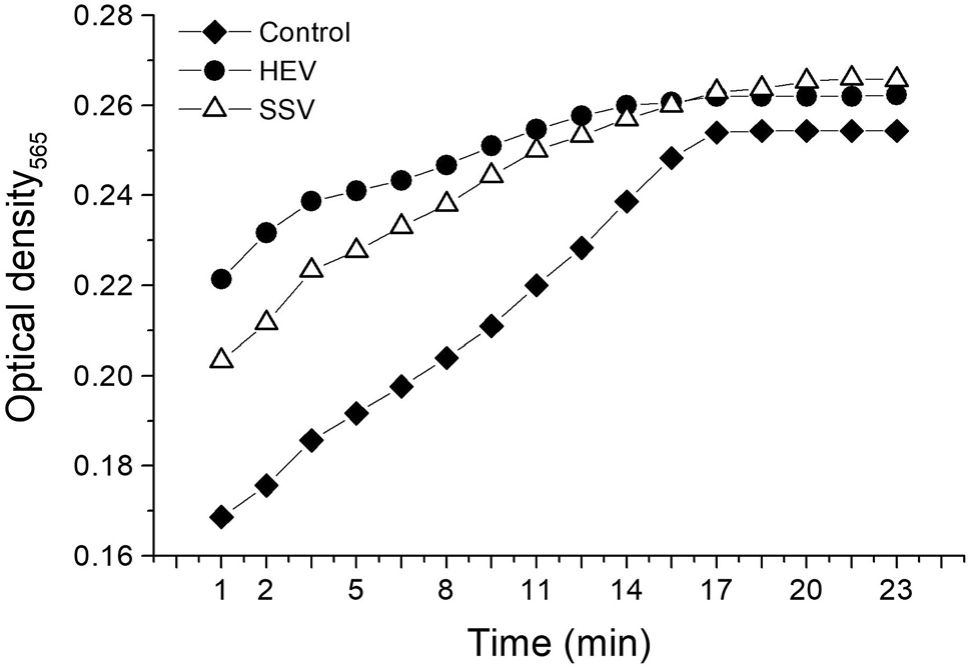
Human plasma coagulation activity of HEV and SSV. The effect of HEV and SSV activity on human plasma coagulation was determined by the re-calcification time assay, as explained in the text. Reaction was followed during 23 min at 37 °C and monitored in a microplate reader at 565 nm.

*M. opercularis* HEV and SSV extracts plasma recalcification assay revealed that extracts lacked thrombin-like activity (plasma did not clot in Ca^2+^ absence). Both extracts displayed a pro-coagulant effect by shortening the clotting time (from minute 1) of human plasma, compared with untreated control (coagulation initiated at minute 7) (Fig. 4).

## Discussion

The term erucism, also called lepidopterism, refers to an injury caused by contact with the venom bristles after adult or larval skin contact. It relates to a typical dermal medical condition caused from one out of 12 butterflies and moths venom species known to harm humans. From North America, 3484 cases of *M. opercularis* caterpillar stings were reported to the Texas Poison Center Network in Texas, USA, during 2000–2016, being the highest number of cases during July (12%) and October–November (59%). Thus showing that this caterpillar has two generations within a year. Patients mostly reported that the sting occurred at their own residence (91% of patients) and were treated outside of a healthcare (89% of patients), where clinical signs mainly involved the dermis (90% of patients), presenting irritation and local pain, wound, erythema, and edema. About 10% presented nausea, vomiting, abdominal pain, numbness, dizziness, headache, and fever. Treatments included washing by irrigation, applying tape to the sting site and pulling it off (to remove spines), and applying ice packs and baking soda, in addition to antihistamines, corticosteroids, or analgesics administration^22^.

After investigating how a disturbance affects *M. opercularis* abundance*,* using netting to cover live oak trees (*Quercus virginiana*) to exclude urban pest birds in Texas, results demonstrated its increase in more than 7,300% on netted versus non-netted trees. This result emphasizes the ecological alteration consequences among species interactions, the potential risk on human health, and the need of considering ecology disturbance under urban planning strategies^23^. One case was a 14-month-old boy who presented symptoms related to this species sting (*M. opercularis* venom causes a painful and pruritic cutaneous reaction). The patient recovered after washing the affected area and antihistamine administration, but was admitted again to the Care Center with lepidopterism after visiting a park. Special care should be implemented with atopic and asthmatic patients, since although rare, anaphylactic reactions to the venoms have been reported^24^.

In South America, after analyzing the progress of pain complaint and its outcome by erucism caused by larvae of several moths (*Automeris* spp., *Hylesia* spp., *Lonomia* spp., *Megalopyge* spp., and *Podalia* spp.), testing five different analgesic schemes among stringed patients admitted in the emergency department in Campinas (southeastern Brazil), Branco et al.^25^ reported that the genus related to most erucism cases was *Podalia*, and that the combined use of local anesthesia plus oral analgesics improved management of severe pain among 278 treated patients. Most reports of envenomation caused by the genus *Megalopyge* in French Guiana, South and North America are related to *M. opercularis*. Nevertheless, three sting cases involving caterpillars of the genus *Megalopyge* reported there were identified as a different species. Clinical symptoms from three patients reporting sharping intense pain (injection of local anesthetic was required), leading to mobility limitation for two of the patients, were not associated with a visible skin lesion. Moreover, patients’ recovery occurred in a few hours, without side effects. From photographs of the caterpillar involved in one case, *M. albicollis* was suspected to be the responsible species^26^. However, involved species in our study was identified as *M. opercularis*.

Due to the multiple reports of affected people by similar larvae within mid-November 2015 and October 2016, and because of the biotechnological interest of caterpillar venom biomolecules^17^, the present study was aimed to identify the endemic venomous caterpillar collected in our region and evaluate some HEV and SSV biological activities on murine and human cells. This represents the first evaluation of their pharmacological and biotechnological potential to date.

HEV and SSV extracts were evaluated for cytotoxicity activity against murine L5178Y-R lymphoma cells and normal resident lymphocytes proliferation, as well as pro-inflammatory cytokine production by hPBMC and human plasma pro-coagulation activity. Heinen & Veiga reported cytotoxic potential of several compounds produced by caterpillars^16^. They mainly reported the role of cecropins, a group of peptides first isolated from the hemolymph of the giant silk moth *Hyalophora cecropia* L. (Lepidoptera: Saturniidae), which possess antitumor and antimicrobial activities^20^. In addition, the “fire caterpillar” *Lonomia obliqua* Walker (Lepidoptera: Saturniidae) venom produces antimicrobial peptides and antitumor activity enzymes (mainly hyaluronidases and phospholipases)^27^. Since resistance against conventional chemotherapy increases, cationic anticancer peptides, as those produced by insects are feasible alternatives^28^.

*M. opercularis* extract has proteolytic activity^29^. After skin contact, its hollow spines release an uncharacterized venom^7^. Therefore, *M. opercularis* is being considering a phanerotoxic species, since it produces and releases its venom throughout spines or setae, whereas among cryptotoxic species, the produced venom remains in the hemolymph^11^. The spines and the hemolymph venoms have similar compounds due to the spines proximity with the hemolymph and the lack of barriers to separate them^11,30,31^. Lepidopteran spines extracts may contain a wide variety of active compounds, such as the storage protein (assembled from six ∼80 kDa polypeptide subunits) that represents the major component of hemolymph^32^.

We observed that HEV and SSV extracts were cytotoxic against L5178Y-R murine lymphoma cells by the mechanism of apoptosis (Fig. 1), but stimulated proliferation of normal lymphocytes (Fig. 2). In this regard, Heinen et al.^15^ showed cytotoxic effect after treating Chinese hamster fibroblasts (V-79) with *Lonomia obliqua* caterpillars crude venom extracts for 24–48 h, at concentrations ranging from 5 to 450 µg/mL, as measured by the MTT colorimetric reduction assay. This work supported concentrations, times, and viability staining procedure used in the present study.

In addition, HEV and SSV extracts showed potential to stimulate IL-1β, IL-6, IL-8, and TNF-α by hPBMC (Fig. 3). Cytokines are known to regulate host immune responses to infection, cancer, and tissue damage. Although IL-1β, IL-6, IL-8, and TNF-α are pro-inflammatory cytokines serving as immune response activators, when they are overexpressed as in an acute response, their effects are exacerbated causing fever, inflammatory processes, tissue destruction, and even death by anaphylactic shock^33^.

On the other hand, thrombosis is considered a major cause of mortality nowadays^34^. The coagulation process is regulated by inhibitors that control clot formation and thrombosis, which may be altered by increased pro-coagulant activity or production of inhibitors^35^. Considering that the hemorrhagic syndrome resulting from *L. obliqua* sting, venom may contain both fibrinolytic and procoagulant activities. In this regard, Veiga et al*.*^13^ found that in addition to the procoagulant activity related to prothrombin activation, *L. obliqua* venom contains at least one fibrinogen/fibrinolytic activity, thus authors suggested the potential use of *L. obliqua* venom as anti-thrombotic agent. Since coagulation alterations have been reported by Lepidoptera’ toxins^3^, in the present study the pro-coagulation effect of HEV and SSV extracts on human plasma was evaluated, showing strong coagulation properties (Fig. 4). Similarly, two species of *Lonomia* caterpillars, *L. obliqua* and *L. achelous* Cramer have been reported to produce toxins that may lead to potentially fatal coagulation activity^3^.

In conclusion, *M. opercularis* HEV and SSV extracts showed antitumor activity against L5178Y-R murine lymphoma cells, involving apoptosis as the cell death mechanism, but stimulated murine proliferation of normal thymus lymphocytes. However, additional experiments using other tumor cell lines are needed to elucidate their full antitumor potential. In addition, HEV and SSV extracts induced production of the pro-inflammatory cytokines IL-1β, IL-6, IL-8, and TNF-α by human PBMC. Furthermore, the extracts showed a potent pro-coagulant effect on human plasma. Overall, results demonstrated that *M. opercularis* HEV and SSV extracts possess anti-tumor, pro-inflammatory, and pro-coagulant activities. This study represents an initial exploration on the bioactivity of *M. opercularis* HEV and SSV venoms, whose results demonstrated their potential for biomedicine application.

## Methods

### Ethical statement

All methods involving human samples were performed in accordance with Institutional guidelines and regulations. Volunteers donating blood samples for experiments in this study provided a signed informed consent and remained anonymous. The donor sample consent informs and the assay involving human samples were reviewed and approved by the Institutional Ethics Committee at Autonomous University of Nuevo Leon (UANL). Experiments related to the use of animals were reviewed and approved by the Institutional Committee for Research Ethics and Animal Welfare of “The College of Biological Sciences” (CEIBA) at UANL with application number CEIBA-2017-005, following Mexican regulation NOM-062-ZOO-1999 entitled *Technical Specifications for the Production, Care and Use of Laboratory Animals*, normative that aligns with the guidelines and basic principles in the NIH Guide for the Care and Use of Laboratory Animals. In addition, standard ethical guidelines for ascites tumor induction in mice and rats^36^ were followed for experiments involving tumor cells obtained from tumor-bearing mice.

### Reagents, culture media, and tumor cell line

Penicillin–Streptomycin solution, and RPMI 1640 and AIM-V media were obtained from Life Technologies (Grand Island, NY). Fetal bovine serum (FBS), Actinomycin D, dimethyl sulfoxide (DMSO), and 3-[4,5-dimethyl thiazol-2-yl]-2,5-diphenyltetrazolium bromide (MTT) were purchased from Sigma-Aldrich (St. Louis, MO). Taq & Go Master Mix 5X, pGEM-T Easy plasmid, and all molecular biology reagents were obtained from Promega (Madison, WI). Oligonucleotides were synthesized by Integrated DNA Technologies (UNIPARTS S.A., Monterrey, N.L., Mexico).

The tumor cell line L5178Y-R (mouse DBA/2 lymphoma) was obtained from The American Type Culture Collection (Rockville, MD), and maintained in culture flasks with RPMI 1640 medium supplemented with 10% FBS, 1% L-glutamine, and 0.5% Penicillin–Streptomycin solution (referred as complete RPMI 1640 medium) at 37 ºC, in a humidified atmosphere of 5% CO_2_ in air. Cellular density was kept between 10^5^ and 10^6^ cells/mL.

### Animals and tumor intraperitoneal implantation

Six- to eight-week old BALB/c female mice were purchased from Harlan Mexico S.A. de C.V. (Mexico, D.F.). Regarding housing conditions, up to five animals per cage were kept in a pathogen- and stress-reduced environment at 24 °C, under a light–dark cycle (light phase, 06:00–18:00 h) in a One Cage 2100 System (Lab Products, Inc., Seaford, DE) and given water and food ad libitum^36^. Three mice were used for L5178Y-R lymphoma induction, which was performed by intraperitoneal (*i.p.*) administration of 0.2 mL of L5178Y-R tumor cells suspension (5 × 10^6^ cells/mouse). After 13 d inoculation, mice were euthanized by cervical dislocation and peritoneal cavity ascites was collected. The ascites suspension was placed in a 50 mL tube containing 10 mL PBS for in vitro cytotoxicity assays^37^.

### Insect source and rearing conditions

Venomous caterpillars were collected from the escarpment live oak *Quercus virginiana* var. *fusiformis* Mill. (Fagaceae) trees growing in the Cumbres National Park of Sierra Madre Oriental, in Monterrey, Nuevo Leon, located northeastern México at 25° 42′ 28.8″ N and 100° 22′ 11.4″ W. Insects collection was performed with a collaboration of Biological Science College (UANL) and the Environmental Education Program of the Wild-Life Cumbres National Park of Nuevo Leon State (*Parques y Vida Silvestre*, https://www.nl.gob.mx/servicios/programa-de-educacion-ambiental). Collected larvae and escarpment live oak leaves were placed inside of a 2-L glass jar with a 2 cm × 2 cm open square metallic cap, covered with wire mesh screen for air exchange. Collected material was transported to the laboratory for larval rearing. Jars with larvae and leaves were incubated at 25  ± 2 °C, 65% ± 5% relative humidity, and 16:8 h light:darkness cycles, inside of a rearing insect room. Larvae were fed on fresh escarpment live oak leaves, previously rinsed in tap water for 30 s. Incubated larvae were tested after reaching the fourth instar. Extra reared larvae were kept feeding until reaching the pupa stage, followed by adults’ emergence, in order to generate and maintain new insect colonies for further experiments.

### Caterpillar venom molecular identification

DNA from three fourth instar caterpillar larvae was extracted, using the Wizard Genomic DNA Purification Kit (Promega) and following the isolating genomic DNA from tissue culture cells and animal tissue protocol. DNA extract was used as a template for PCR amplification of specific primers for the cytochrome oxidase subunit (COI) F1 5′AAC WYT ATA YTT TAT TTT TGG 3′ R and 5′TGT TGR TAW ARR ATW GGR TC 3′, designed from Genbank *Megalopyge* genus sequences.

PCR was performed using GoTaq Green Master Mix (Promega) in a 50 µL volume, with 100 ng of DNA as template and 1 µM of forward and reverse primers. Thermal cycling conditions included an initial denaturation step at 94 °C for 10 min, followed by 35 cycles of denaturation at 94 °C for 40 s, annealing at 60 °C for 40 s, and elongation at 72 °C for 2 min.

Amplified PCR products were ligated into pGEM-T Easy (Promega) in competent *E. coli* TOP-10 cells. Detected plasmids were purified using the Wizard Plus SV Minipreps DNA Purification System. Sanger sequencing was performed with standard vector M13F and M13R primers by the *Instituto de Biotecnología* at *Universidad Nacional Autónoma de México*. The sequence obtained was analyzed on platform Boldsystem.

### HEV and SSV spine setae samples

HEV was obtained by performing a puncture on the third false leg from each larva head. Released fluid (~ 200 µL) was collected and centrifuged at 9,600 rpm for 2 min. The resulting supernatant protein content was quantified on a NanoDrop Lite kit and adjusted to 1 mg/mL. This was used as a stock for further dilution and dosage preparations^38^. In addition, SSV was obtained from four reared fourth instar venomous caterpillars, extracted according to da Silva et al.^39^. Spine setae were cut from the caterpillars’ integument, homogenized, sonicated in sterile PBS, and processed as described for HEV.

### HEV and SSV cytotoxicity against murine L5178Y-R lymphoma cells

To determine the direct in vitro effect of HEV and SSV on tumor cell growth, L5178Y-R cell suspensions (from *i.p.* lymphoma grown in female BALB/c mice as explained above) were adjusted to 5 × 10^4^ cells/mL in complete RPMI 1640 medium. We evaluated the antitumor effect of a broad range of concentrations of HEV and SSV, following the cytotoxicity assay previously described^15^. One hundred microliters of the cell suspensions were then added to flat-bottomed 96-well plates (Becton Dickinson, Lincoln Park, NJ), containing triplicate cultures (100 µL) of complete RPMI 1640 medium (unstimulated control), HEV or SSV (7.8–500 µg/mL)^37^, using 3.1–125 µg/mL Vincristine (Sigma-Aldrich), as positive control. After incubation for 44 h at 37 °C in 5% CO_2_, MTT (0.5 mg/mL, final concentration) was added, and cultures were incubated for additional 4 h. Cell cultures were then incubated for 16 h with 100 µL DMSO to dissolve formazan crystals, and optical densities (ODs) were read in a microplate reader (Bio-Tek Instruments, Inc., Winooski, VT) at 540 nm^37^. Percentage of cytotoxicity was calculated as follows:

$$ \% {\text{ Cytotoxicity}}\, = \,{1}00 - \left[ {\left( {{\text{OD}}_{{{54}0}} {\text{in HEV{-} or SSV{-}treated cells}}/{\text{OD}}_{{{54}0}} {\text{in untreated cells}}} \right)\, \times \,{1}00} \right]. $$

The Statistical Package for the Social Sciences version 17.0^40^, was used to calculate the inhibitory concentration at 95% (IC_95_), selecting the Probit analysis.

### Apoptosis assay

Cellular death type resulting from HEV- or SSV-mediated L5178Y-R cytotoxicity was determined according to Reyna-Martínez et al.^41^. For this, 3 × 10^6^ cells were exposed to HEV or SSV IC_50_ using flat-bottomed, 24-well plates (Becton Dickinson), and incubated for 24 h under the same conditions as for the cytotoxicity assay. Treated cells were aliquoted into microtubes, washed by centrifugation at 9,600 rpm (Sorvall ST16R Centrifuge; ThermoScientific, Pittsburgh, PA), and suspended in 500 μL of complete RPMI 1640 medium. Cells were then stained adding 1 μL of 100 μg/mL acridine orange and 1 μL of 100 μg/mL ethidium bromide, and incubated for 5 min. Next, cultured cells were washed three times by centrifugation 9600 rpm with 1 mL PBS and suspended in 100 μL of PBS 1×, after which 10 μL of cell suspension samples were observed in a fluorescence microscope adapted with a rhodamine filter (540–570 nm), using Actinomycin D (800 ng/mL) as positive control.

Uniform green stained cells were quantified as viable cells and spotty green or granular core cells were quantified as in early apoptosis. Orange dots or cells with large granules similar to those observed in early-apoptosis cells were quantified as in late apoptosis, whereas uniform orange hue cells were quantified as in necrosis^42^.

Staining cells results were validated by the DNA degradation method^41^, where DNA like-ladder fragmentation indicates apoptotic activity, whereas DNA smear represents cell death by necrosis. DNA extracted from 1 × 10^6^ cells per treatment were tested using the AxyPrep Multisource Genomic DNA Miniprep kit (Axygen) in 1% agarose gel electrophoresis at 100 V for one hour. The gel was then stained with 5 ng/mL ethidium bromide and analyzed on a GelDoc XR photo-documenter (Bio Rad, Berkeley, CA).

### Lymphocyte proliferation assay

The effect of venom caterpillar HEV and SSV extracts on murine lymphocyte proliferation was determined by the MTT reduction colorimetric technique^37^. Two mice were euthanized and thymuses were immediately removed after mice death, a single cell-suspension was prepared by disrupting the organs in RPMI 1640 medium, as previously reported^37^. Cell suspensions were then washed three times in this medium, suspended, and adjusted to 1 × 10^7^ cells/mL in complete RPMI 1640 medium. One hundred microliters of thymus cell suspensions were added to flat-bottomed 96-well plates (Becton Dickinson) containing triplicate cultures (100 µL) of complete RPMI 1640 medium (unstimulated control), HEV and SSV at 7.8, 15.6, 31.25, 62.5, 125, 250, and 500 µg/mL^15,37^, and the positive control Concanavalin A (6.25 μg/mL) for 48 h at 37 °C in 95% air-5% CO_2_ atmosphere. After 44 h of incubation, MTT (0.5 mg/mL, final concentration) was added, and cultures were incubated for additional 4 h. Cell cultures were then incubated for 16 h with 100 µL of DMSO and ODs, resulting from dissolved formazan crystals, were then read in a microplate reader (DTX 880 Multimode detector, Becton Dickinson, Austria) at 570 nm^37^. To calculate the lymphoproliferation index, the obtained values between the samples were compared. For this, values recorded by extracts treated cells were divided with the value given by Concanavalin A (tested as mouse T-cell mitogen) as follows: OD_570_ in treated cells/OD_570_ in Concanavalin A treated cells. Therefore, all values were compared with the control, where the lowest concentrations have a value of 1, since there was no difference compared with the control.

### Human peripheral blood mononuclear cells (hPBMC) cytokine response to *M. opercularis* extracts

Cytokine production by hPBMC was measured after HEV and SSV extracts exposure. For this, hPBMC were isolated with Ficoll-Paque Plus (GE Healthcare, Uppsala, Sweden) and adjusted to 1 × 10^6^ cells/mL in complete RPMI 1640 medium. One hundred microliters of the cell suspension were placed in a 96-well plate in the presence or absence (untreated control) of 100 μL of HEV or SSV *M. opercularis* extracts at 3.91, 7.81, 15.62, 31.25, 62.5, and 125 µg/mL^15^ in complete RPMI 1640 medium. Plates were then incubated at 37 °C for 48 h and centrifuged at 400 rpm for 5 min.

Cell‐free supernatants were then subjected to IL-1β, IL-6, IL-8, and TNF-α levels determination by cytometric bead arrays (CBA) (BD Biosciences, San Jose, CA) on a BD Accuri C6 Flow Cytometer Sampler (BD Biosciences, Ann Arbor, MI), following manufacturer's instructions, and data analyzed with the FCAP Array v3.0 (SoftFlow Inc.). Results were adjusted by subtracting the basal levels of cytokines from untreated hPBMC (negative control) and data analyzed by Prism 6 software (GraphPad Software Inc., La. Jolla, CA)^43^.

### Coagulation assay

The effect of HEV and SSV activity on plasma coagulation was assessed, using the re-calcification time assay^44^, adapted for a microplate reader. For this, 1 mg/mL HEV and SSV reactive samples were prepared in 20 mM Tris–HCl buffer pH 7.4 and sterilized by filtration with a 0.22 μm micropore filter. Reactive samples consisted of 50 μL of citrated human plasma, 50 μL of HEV or SSV samples at 250 μg/mL (based on the concentration that produced maximal cytotoxicity in lymphoma cells), and 100 μL Tris–HCl buffer to a final volume of 200 μL. They were then incubated for 5 min at 37 °C, after which 10 μL of 150 mM CaCl_2_ were added for coagulative process re-activation, following the reaction during 23 min at 37 °C, and ODs were read in a microplate reader (Bio-Tek Instruments, Inc.) at 565 nm.

### Statistical analysis

Results were expressed as means ± SD of triplicate determinations from three independent experiments. Statistical significance (*p* ≤ 0.05) was assessed by one-way analysis of variance and by the Student’s *t* test.

## Supplementary information

Supplementary Information 1.

## Data Availability

All data generated or analyzed during this study are included in this published article (and its Supplementary Information file).
