# Supplementary figures and images for: In vitro antitumor, pro-inflammatory, and pro-coagulant activities of Megalopyge opercularis J.E. Smith hemolymph and spine venom
Source: Sci Rep. 2020 Oct 27;10:18395. doi: 10.1038/s41598-020-75231-1 (PMC7592054; doi:10.1038/s41598-020-75231-1)

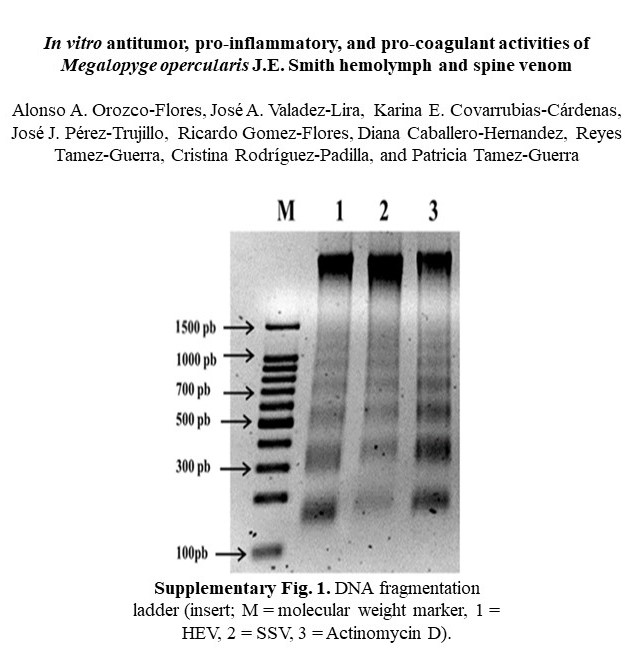

Supplement: Supplementary file 1 — Supplementary Information 1. [file 41598_2020_75231_MOESM1_ESM.jpg]
